# Application of the CRISPR/Cas9 System to Drug Resistance in Breast Cancer

**DOI:** 10.1002/advs.201700964

**Published:** 2018-04-15

**Authors:** Yinnan Chen, Yanmin Zhang

**Affiliations:** ^1^ School of Molecular Sciences Arizona State University Tempe AZ 85287 USA; ^2^ School of Pharmacy Health Science Center Xi'an Jiaotong University Xi'an Shaanxi Province 710061 P. R. China; ^3^ State Key Laboratory of Shaanxi for Natural Medicines Research and Engineering Xi'an 710061 P. R. China; ^4^ Shaanxi Institute of International Trade & Commence Xianyang 712046 P. R. China

**Keywords:** breast cancer, CRISPR/Cas9, drug resistance, drug therapy, reverting resistance

## Abstract

Clinical evidence indicates that drug resistance is a great obstacle in breast cancer therapy. It renders the disease uncontrollable and causes high mortality. Multiple mechanisms contribute to the development of drug resistance, but the underlying cause is usually a shift in the genetic composition of tumor cells. It is increasingly feasible to engineer the genome with the clustered regularly interspaced short palindromic repeats (CRISPR)/associated (Cas)9 technology recently developed, which might be advantageous in overcoming drug resistance. This article discusses how the CRISPR/Cas9 system might revert resistance gene mutations and identify potential resistance targets in drug‐resistant breast cancer. In addition, the challenges that impede the clinical applicability of this technology and highlight the CRISPR/Cas9 systems are presented. The CRISPR/Cas9 system is poised to play an important role in preventing drug resistance in breast cancer therapy and will become an essential tool for personalized medicine.

## 1 Introduction

Breast cancer is one of the main causes of cancer‐related death in women worldwide, accounting for an estimated 28% of new cancers.^1^, ^2^, ^3^ It is a highly heterogeneous disease, and multiple signaling pathways can mediate its initiation and progression.^4^ According to gene expression profile studies, different subtypes of breast cancer have been identified based on the expression of estrogen receptor (ER) and/or progesterone receptor (PR), and human epidermal growth factor receptor 2 (HER2). ER belongs to the superfamily of nuclear receptors,^5^ which played a critical role for development and progression of breast cancer. ER^+^ disease is one of the most common types of breast cancer, accounting for nearly 70–75% of all cases.^6^ There are two different molecular forms of the ER, which are ERα and ERβ, coded by different genes, and their expression patterns differ.^7^ The ER pathway is targeted by endocrine therapies that either repress ER functions or deplete the ligand estrogen, its ligand. While endocrine therapies are very effective, de novo and acquired resistance frequently occurs over the course of therapy.^8^ The drugs used in the treatment of breast cancer include pharmacological agents for endocrine therapy, such as drugs that target HER2 and other signal molecules, and conventional chemotherapy. The detailed representative drugs of conventional chemotherapy for breast cancer in clinic are listed in **Table** **1**.

**Table 1:** Overview of anticancer drugs for breast cancer

Class	Target	Compounds	Mechanism of action	Ref.
Drugs in targeted therapy	ER	Anastrozole	Binding reversibly to the aromatase enzyme through competitive inhibition, inhibiting the conversion of androgens to estrogens	[17,18]
		Exemestane	Disrupting estrogen signaling by irreversible and inactivating binding to the aromatase enzyme, and significantly reducing estrogen biosynthesis and intratumoral levels of estrogen	[18]
		Fulvestrant	Competitive binding of ER and ER antagonist, preventing its dimerization and facilitating its proteasomal degradation	[19]
		Goserelin	Suppressing FSH and LH secretion to menopausal levels, reducing estrogen and progesterone production	[20]
		Letrozole	Aromatase inhibitor, disrupting estrogen signaling by reversible and competitive binding to the aromatase enzyme; significantly reducing local estrogen biosynthesis	[18,21]
		Raloxifene	Selective estrogen receptor modulator (SERM) through binding to ERs	[22]
		Tamoxifen	Competitive inhibitor of estrogen binding to the ER	[13]
		Toremifene	SERM	[3,23]
	HER2‐enriched	Antibody‐drug conjugate trastuzmab emtansine	Inhibiting HER2 signaling and disrupting dynamics of microtubules	[24,25]
		Lapatinib	Tyrosine kinase inhibitor (TKI) of EGFR/HER1 and HER2, blocking of the ATP‐binding site in the cytoplasmic domain of HER2, which leads to inhibition of signal transduction cascade from the receptor	[25–27]
		Pertuzumab	Anti‐HER2 mAb, binding to a different HER2 domain and inhibiting dimerization	[27,28]
		Trastuzumab	Blocking the extracellular part of the bond to the HER2 receptor ligand and inhibiting the pathological signal of HER2	[12,29]
	TKI	Dasatinib	A TKI targeting to various kinases, such as Src, BCR‐Abl, FAK, c‐Kit, and hormone receptor positive breast cancer	[30]
		Iniparib	Irreversibly inhibiting PARP1 and possibly other enzymes through covalent modification	[2,31]
		Neratinib	Pan‐ErbB TKI, inhibiting HER4 as well as HER1/EGFR and HER2	[2,32]
		Olaparib	Poly‐(ADP‐ribose) polymerase (PARP) inhibitor	[33]
Drugs in conventional chemotherapy	No specific and broad‐spectrum drugs	Actinomycin D	Inhibiting transcription by binding DNA at the transcription initiation complex and preventing elongation of the RNA chain by RNA polymerase	[34]
		Bleomycin	Inducting DNA strand breaks, inhibiting incorporation of thymidine into DNA strands	[35]
		Cyclophosphamide	Interfering mainly in DNA replication by its metabolite phosphoramide mustard and irreversibly leading to cell apoptosis	[36]
		Carboplatin	Binding mainly to DNA	[37]
		Cisplatin	Interfering with DNA replication	[38]
		Capecitabine	Prodrug of 5‐FU, alternative antimetabolite, and thymidylate synthase inhibitor (inhibiting the synthesis of thymidine monophosphate)	[39,40]
		Doxorubicin	Interacting with DNA by intercalation, affecting DNA enzymes, inhibition of macromolecular biosynthesis, and inducing cell apoptosis	[41]
		Docetaxel	High cytotoxic activity on all cell types by various mechanisms, such as binding to microtubules reversibly with high affinity	[42]
		Eribulin	Inhibiting microtubule dynamics, triggering apoptosis of cancer cells following prolonged and irreversible mitotic blockade	[43]
		5‐Fluorouracil	Principally inhibiting thymidylate synthase	[44]
		Hydroxycamptothecine	Binding to Topo I and DNA complex (the covalent complex), inhibiting the topo I and inducing apoptosis	[45]
		Ixabepilone	Enhancing microtubule stability and formation of abnormal mitotic spindles, which induce G2‐M cell cycle arrest and apoptosis	[46,47]
		Methotrexate	Inhibiting synthesis of DNA, RNA, thymidylates, and proteins	[48]
		Nab‐paclitaxel	Active transport across endothelial cells via the gp60/caveolin‐1 receptor pathway, active binding of albumin–paclitaxel complexes by SPARC, targeting HER2	[49]
		Paclitaxel	Antimicrotubule agents, inhibiting disassembly of microtubules	[50]
		Trabectedin	DNA‐interacting agent and transcription inhibitor, downregulating P‐glycoprotein/MDR1 by immunomodulation	[46,51]
		Vinorelbine	Alternative anti‐microtubule agent, inhibiting mitosis through interaction with tubulin	[39,52]

Although there are numerous therapeutic options available for patients with breast cancer, allowing for a number of approaches designed to inhibit ER or estradiol synthesis, including sequentially alternating drugs for long‐term inhibition of estrogen signaling, drug resistance is a problem for most therapies.^6^, ^9^ Drug resistance renders breast cancer uncontrollable and causes high mortality, with more than 90% of unsuccessful treatments due to acquired resistance and multidrug resistance (MDR).^10^, ^11^ For instance, tamoxifen, a selective ER modulator, is the most successful treatment for ER^+^ cancer. It reduces the risk of recurrence after five years by 41% and mortality by 34%.^12^ However, despite its widespread use and success, over 40% of ER^+^ patients with advanced disease failed to respond effectively to tamoxifen. Even though those who responded at the beginning of treatment eventually developed acquired resistance. Thus, overcoming resistance to drugs targeting ER signaling remains an unmet need in clinical breast oncology.^13^

It is extremely time‐consuming and costly to develop new drugs with different targets to avoid the known mechanisms that cause resistance. Therefore, in recent years, a large body of work has focused on understanding the underlying mechanisms leading to resistance, and additional therapies have been developed to prevent acquired resistance. Clustered regularly interspaced short palindromic repeats (CRISPR)/associated (Cas)9 is a gene‐editing technology, which can correct errors in the genome and switch on or off certain genes in cells and organisms fast, cheaply, and relative easily. It has already been reported that CRISPR/Cas9 can be used to repair defective DNA in animal models, such as mice, resulting in the cure of genetic disorders, and it has been showed that human embryos can be modified.^14^, ^15^, ^16^

This review discusses how CRISPR/Cas9 may be used to solve issues related to breast cancer drug resistance by reversing resistance gene mutations, resistance target screening, and identification of drug therapy in breast cancer. Furthermore, we specifically discuss efficiency, modification of the protospacer adjacent motif (PAM), targeting selection, and off‐targets in the application of CRISPR/Cas9.

## 2 CRISPR/Cas9

### 2.1 The CRISPR/Cas9 System

In eukaryotic genomes, the billions of DNA bases are hard to manipulate, but progress in genome editing has motivated the investigation of new anticancer targets and development of novel therapeutics. Homologous recombination (HR) targets exogenous repair templates that contain a sequence homology to the donor site. A series of studies have shown that targeted DNA double‐strand breaks (DSBs) significantly stimulate genome editing through homology‐directed repair (HDR) events.^53^ In the absence of an exogenous homology repair template, the error‐prone nonhomologous end joining (NHEJ) repair pathway can lead to insertion or deletion mutations at localized DSBs.^54^ HR‐mediated targeting has facilitated the creation of knock‐in and knock‐out in animal models by manipulation of germline competent stem cells. These studies on genome editing have established powerful ways to modify eukaryotic genomes. These processes permit highly precise alterations, nevertheless, the desired recombination events occur extremely infrequently (only 1 out of 10^6^–10^9^ cells), which is huge challenge for large scale application of gene‐modifying experiments.^53^

At present, there are four major classes of customizable DNA‐binding proteins that have been used and engineered for effective genome editing through the introduction of DSBs at specific site DNA: meganucleases from microbial mobile genetic elements, zinc‐finger nucleases from eukaryotic transcription factors, transcription activator‐like effectors from bacteria, and most recently, the RNA‐guided DNA endonuclease Cas9.^55^

**Figure 1:**
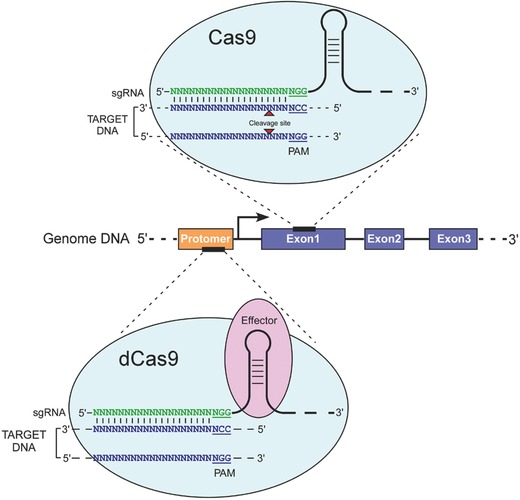
Schematic illustration of genome engineering using the CRISPR/Cas9 system. Top: The *Streptococcus pyogenes*‐derived CRISPR/Cas9 RNA‐guided DNA endonuclease can recognize a coding exon of a gene of interest (blue) via a sgRNA sequence. sgRNA can anneal to a specific target sequence adjacent to a PAM sequence in the form of NGG or NAG. Cas9‐mediated induction of a DSB (red arrows) in the DNA target sequence leads to indel mutations via NHEJ or precise gene modification via HDR. Bottom: Catalytically inactive dCas9 can target promoters or enhancers of genes of interest (orange). Chimeric sgRNAs containing aptamers can bind to RNA‐binding domains fused to effector domains, such as transcriptional activators/repressors, chromatin modifiers, or fluorescent proteins (purple).

The most cutting‐edge genome‐editing technology, CRISPR/Cas9, simplifies the recognition step by using a short RNA guide, which associates with the Cas9‐binding protein. The system consists of two core biological components: the RNA guided DNA endonuclease, Cas9, and a chimeric single‐guide RNA, sgRNA. The sgRNA contains both a CRISPR RNA component (crRNA) as well as a trans‐activating crRNA, that binds to Cas9 and directs it to a specific sequence of interest via Watson–Crick base pairing (**Figure** **1**).^56^ Multiplexed targeting by endonuclease Cas9 can be achieved at an unprecedented scale by employing a battery of short guide RNAs instead of a library of bulky proteins. The only criterion for defining the target is that it needs to be close to a PAM, DNA sequence, consisting of either NGG or NAG.^57^ The Cas9‐induced DSB activates the DNA repair machinery through either the NHEJ pathway for indels or HDR for precise modification in presence of a donor DNA template. By simply combining Cas9 with a complementary sgRNA to a targeted DNA sequence, researchers can achieve high cleavage efficiency of the gene. The ease of the Cas9 targeting, its relatively high efficiency as a site‐specific endonuclease, and the possibility for high multiplexed modification opened up a broad range of applications in biotechnology and medicine.

### 2.2 CRISPR/Cas9‐Based Effectors

In addition to gene knockout that is mediated by the error‐prone repair of targeted DSBs, catalytically inactive Cas9 (dCas9) can be coupled with a transcriptional activator or repressor to modulate gene expression.^58^, ^59^ dCas9 itself has a repressive effect on gene expression because of steric hindrance of the transcription initiation components. Chromatin modifying repressor domains have been fused to dCas9 to improve the repression effect.^60^ Additionally, scaffold RNAs fused to an RNA aptamer can be used to recruit activator or repressor effectors.^61^ The effector systems are employed to model gene expression alterations and copy number variations in eukaryotic cells. These CRISPR/Cas9‐based effector systems also provide an advantage, particularly in studying cancer‐associated trans‐acting or cis‐acting regulatory noncoding RNAs, as well as modifying endogenous gene expression (Figure 1).^62^ Alternatively, dCas9 has been used to image genomic loci in living cells.^63^ The dCas9‐based transcriptional suppression and activation systems are referred to as CRISPRi and CRISPRa, respectively.^59^ Although dCas9 mediated repression and RNA interference (RNAi)‐based tools seem to result in a similar molecular effect, dCas9 repression occurs by inhibiting transcription instead of silencing mRNAs in the cytoplasm, which might result in varying cellular responses.

## 3 The Superiority of CRISPR/Cas9 in Solving Breast Cancer Drug Resistance

The conventional way to minimize acquired drug resistance is to combine agents with different targets. However, the mechanisms of action of each drug interact with each other, and the effect is hard to predict. Still another solution is to improve the specificity of the anticancer agent to decrease the possibility of acquired drug resistance, especially for cases of MDR. Additionally, blocking or reversing resistance factors would permit the reuse of existing anticancer drugs.

The development of cancer, including that of breast cancer, is a multistep and complicated process arising from a series of genetic events. Genome sequencing studies of multiple cancer types have revealed myriads of point mutations, copy number alterations, and chromosome rearrangements.^64^ Many approaches in vivo and in vitro have been used to validate oncogenes, as well as drug‐resistant genes. These methods can be classified into two groups: loss‐of‐function (e.g., RNAi) and gain‐of‐function (cDNA‐based over‐expression) of the gene of interest. Although these approaches have played an important role in many significant discoveries in cancer biology over the past decades, they have some crucial limitations.^65^ cDNA‐based expression systems may bring to supraphysiological levels of gene expression, which can cause artifact effects on cell biological processes. Moreover, knockdown of a gene of mRNA levels by RNAi is incomplete and the remaining mRNAs may still play a functional role.^66^ This can prevent identification of targets that require mRNA complete inactivation. Genome engineering in mouse or human cell models is more complete; however, it has been technically challenging and time consuming for the traditional approaches. The Cre/LoxP system has been the main method used in modeling many types of cancers in mice.^67^ Given the complexity of genetic events in cancer, it has been challenging to functionally interrogate the role of each mutation or the combinatorial effect on genes involved in tumorigenesis.^65^

The use of the CRISPR/Cas9 system offers a fast approach for targeted modification of endogenous loci, overcoming limitations of the other methods mentioned above. For example, this system could be used in modeling of genetic variants, somatic genome engineering, and CRISPR‐based effector regulation or genetic screening in vitro or in vivo.^15^, ^65^ Over the last few years, numerous studies have reported efficient gene disruptions or modifications in a variety of cancer cells via CRISPR/Cas9‐mediated NHEJ or HDR. It has been successfully used in gene editing of several sites within the mammalian genome of established cell lines, as well as patient‐derived xenografts to engineer indel mutation, modification, or chromosomal rearrangements.^62^, ^68^ With the developments in CRISPR/Cas9‐based genome editing, many of the challenges in generating somatic or germline mutations have become trivial.

## 4 How CRISPR/Cas9 Overcomes Drug Resistance

**Figure 2:**
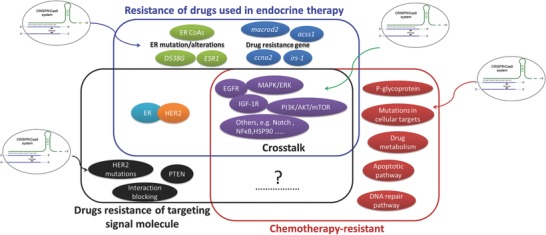
Schematic representation of several possible mechanisms involved in drug resistance in breast cancer therapy. This mainly includes drug resistance analysis of pharmacological agents used in endocrine therapy and targeted signaling molecules, and chemotherapy resistance. The blue rectangle refers to the section of endocrine therapy, black rectangle refers to the section of targeting signaling molecules, and red rectangle refers to the section of chemotherapy‐resistance. The crosstalk is what is in common of the three kinds of drug resistance mechanisms, which has a complicated network and is responsible for drug resistance. CRISPR/Cas9 can mainly apply to drug resistance based on crosstalk, the target mutation/alteration, and drug resistance genes.

Drug resistance in breast cancer is complex and involves multiple mechanisms (**Figure** **2**). We discuss several key reasons (Notes 1–3) for drug therapy failure in breast cancer, and examine how the CRISPR/Cas9 system overcomes these challenges.

### 4.1 Genome Manipulation to Solve Drug Resistance

Cellular genes that have mutated to oncogenes have a huge potential as targets for human cancer treatment. Drug resistance caused by ER or HER2 mutation/alteration is described in Note 1 (Section 4.1.1). Oncogenes drive cell proliferation and stimulate cell signaling pathways inappropriately. They are usually active in the presence of a wild‐type allele of the proto‐oncogene, therefore they can be claimed to act in a dominant manner.

#### 4.1.1 Note 1: ER and HER2 Mutation/Alteration

*ER Mutation/Alteration*: Clinical clues to understand resistance to endocrine therapy can be related to the loss of ERα expression, ERα mutations, loss of ERβ expression, PR deficiency, and other factors (such as alteration in the metabolism of the drug). There has been a renewed interest in understanding and uncovering genetic effectors of endocrine therapy resistance with the recent discovery of ER mutations and translocations.^13^ Mutation of ER might affect the response to antiestrogens. A recent clinical sequencing study in patients with advanced ER+ breast cancer has identified a D538G mutation within ER in patients resistant to endocrine therapy, causing a change from aspartic acid to glycine at position 538 within the ligand binding domain.^18^ Similar to the D538G mutation, ER has been found to confer constitutive ligand‐independent transcriptional activity that mimicked that of estrogen‐bound wild‐type ER with reduced tamoxifen‐binding affinity. Mechanisms involve expression of truncated isoforms of ER such as ERα36 or other ERR (i.e., ERR gamma, ERRγ), both of which have been associated with a reduced response to tamoxifen.^69^

*HER2 Mutation/Alteration*: HER2 is overexpressed in 25–30% of human breast tumors, which has a predictive role for prognosis in the process of chemotherapy and endocrine therapy.^4^, ^12^, ^70^ The HER2 pathway engages in crosstalk with ER and growth factor receptor pathways and as such has a role in endocrine therapy resistance in breast cancer.

The probability of HER2 mutations is 1.67% in breast cancer.^71^ Mutational activation of HER2 can result from three types alterations: small insertions and missense site mutations in the kinase domain, missense mutations in the extracellular domain, or large deletions of the extracellular domain which yield a truncated HER2.^72^, ^73^ Most mutations are mainly located in the exons (from 19 to 21) of the tyrosine kinase (TK) domain, and are encoded in exons 18–23.^74^ HER2 kinase domain mutations can be classified as: missense point mutations, small in‐frame insertions or duplications mostly occurring in exon 20, and in‐frame deletions. These HER2 mutations are the most common type found in patients lacking HER2 overexpression and most of them were found in the TK domain (seven of these HER2 kinase domain mutations are activating and oncogenic, including G309A, D769H, D769Y, V777L, P780ins, V842I, and R896C).^71^, ^72^

CRISPR/Cas9 system can be used to target the mutated form of the cellular oncogene to disrupt or inactivate it. For instance, the Src family of oncogenes requires tyrosine kinase activity to transform thus it could be targeted by CRISPR/Cas9 directed toward the tyrosine kinase domain.^75^ Tang and Shrager proposed a practical clinical application of the CRISPR/Cas9 system to correct epidermal growth factor receptor (EGFR) mutations in patients with lung cancers. The “personalized molecular surgery” expression plasmid can be packaged into a virus and delivered intratracheally or intravascularly to patients.^76^ Based on the mechanism analysis of drug resistance in breast cancer, a similar strategy could also be used in ER or HER2 mutants in patients with breast cancer. sgRNA will be designed to target specific sequences in the mutated exon of ER or HER2 to repair the mutation(s). The replacement will eradicate the carcinogenic mutation, thus ending the constitutive activity. Meanwhile, CRISPR/Cas9 can also disrupt the specific functional domains of ER or HER2, which are necessary for oncogenic activity, and therefore, lose its acquired drug resistance. This strategy will directly target the cause of the disease in a personalized and permanent manner.

The CRISPR/Cas9 system has the potential to be engineered to provide a specific and efficient approach against many types of oncogenic alterations in cancer cell lines. Conversely, tumor suppressors can become inactivated by the mutations in their genes, and they have a huge potential as targets for treating human cancer by correcting them with CRISPR/Cas9 specifically.

Signaling pathways govern the proliferation of cancer cells. Signaling cascade through complex growth factor receptor pathways, which activate ERs, is emerging as essential causes of endocrine resistance (Note 2, Section 4.1.2). Since these factors play crucial roles in the signaling cascades, we propose that the CRISPR/Cas9 system could be an effective method to revert drug resistance due to crosstalk dysregulation by manipulating ER, HER2, and EGFR. The CRISPR/Cas9 therapy could limit secondary genomic mutations, to a minimal level, which otherwise may result in endocrine therapy resistance, with a careful sgRNAs design and efficient delivery. This approach could be combined with traditional surgery, radiation therapy, and chemotherapy.

#### 4.1.2 Note 2: Crosstalk Dysregulation

Phosphorylation and activation of ERs can be triggered by several intracellular kinases.^77^, ^78^ In particular, ER is phosphorylated at certain residues, including serine 106/107, 118, 167, 305, and threonine 311, residing mainly in the AF‐1 binding domain, as well as in other domains.^78^ Hundreds of new specific agents are in development for targeting several signaling pathways in patients with endocrine resistant breast cancer.^2^ The main crosstalk pathways are EGFR, HER2, phosphatidylinositol 3‐kinase(PI3K)/Protein kinase B (AKT)/mammalian target of rapamycin (mTOR), the insulin‐like growth Factor‐1 receptor (IGF‐1R), and mitogen‐activated protein kinase (MAPK) signaling pathway and Src family tyrosine kinases.

### 4.2 Maintenance of Sensitivity for Drug Therapy of Chemotherapy‐Resistance

The CRISPR/Cas9 system has been proposed as a therapeutic method to overcome drug resistance in chemotherapy‐resistant cancers (Note 3, Section 4.2.1). Blocking resistance factor(s) is an attractive strategy to further use existing anticancer agents. There are several strategies to enhance drug therapy, including altering membrane transport protein to increasing drug efflux, enhancing DNA repair, and detoxification.^79^ Ha et al. tried to overcome doxorubicin‐resistance cancer cells by using the CRISPR/Cas9 system to target MDR1.^80^ MCF‐7/ADR cells were treated with doxorubicin after disruption of MDR1 by Cas9‐sgRNA, and possible drug sensitivity recovery was examined. The potency of doxorubicin was enhanced in the cells treated with CRISPR/Cas9 expression construction using a proper delivery platform.^80^ This result indicates that disruption of this drug resistance‐related gene can be considered to overcome MDR in cancer cells. Another transporter, breast cancer resistance protein encoded by the ABCG2 gene, is associated with an MDR phenotype of MCF7 cells. CRISPR/Cas9 systems (sgRNA and Cas9 expression plasmid and donor DNA plasmid) targeting these genes can be packaged into viruses and injected intratracheally, or intravascularly into patients. Swiech et al. have delivered Cas9 and guide RNAs into the adult mouse brain in vivo using adeno‐associated viral vectors to target multiple genes.^81^ It is feasible to apply a similar strategy to target other types of cancer‐driving genomic changes.

For instance, glutathione‐S‐transferases (GST) catalyzed glutathione conjugation and elevated expressions of levels of the GST‐p subgroup are associated with cisplatin resistance in several types of cancer cells.^82^ Based on the strategy described by Ha et al.,^80^ after identification of the specific expression of genes related to drug resistance mentioned above, they could be edited by the CRISPR/Cas9 system to recover drug sensitivity, which makes downregulation of efflux‐mediated chemotherapy resistance available. This is a promising way to overcome MDR of cancer cells.

#### 4.2.1 Note 3: Chemotherapy Resistance

Cancer cells become resistant to one or more chemotherapeutic agents after repeated treatment, which is the main hurdle to overcome to achieve successful cancer therapy.^11^, ^80^ Recognized mechanisms of chemotherapy resistance include altered expression of the ABC superfamily of transporters, such as P‐glycoprotein encoded by the mdr1 gene;^83^, ^84^ alteration of DNA repair pathways, mutations in cellular targets (i.e., topoisomerase II or tubulin),^85^ resistance to initiation of the apoptotic pathway, and the development of constitutively activated signaling pathways, altering drug metabolism.^11^, ^83^, ^86^ For example, overexpression and/or activation of HER2 confer resistance of cancer cells to chemotherapeutic drugs.^87^

## 5 Resistance Target Screening and Identification

Multiple players within the same mechanism can contribute to cancer drug resistance. Screening and identification of such molecular events may be critical to elucidate the molecular mechanisms inducing resistance to first‐line therapy. Molecular screening of signal pathways regulated in resistant tumor cells could have a major implication in early stage of drug development.^2^ Comprehensive approaches are required to understand the elements that lead to drug resistance.^88^

Previously, loss‐of‐function studies were carried out by using RNAi libraries to identify candidate drivers of resistance.^89^ These studies target at the open reading frame (ORF) with small interfering RNA or short hairpin RNA libraries. RNAi is a conserved natural pathway that is triggered by dsRNA and results in the selective repression of mRNA transcripts with sequence complementarity to one strand of the dsRNA. It has been shown that silencing phosphatase and tensin homolog as well as cyclin‐dependent kinase 10 causes resistance to tamoxifen and trastuzumab, respectively, in breast cancer.^90^

**Figure 3:**
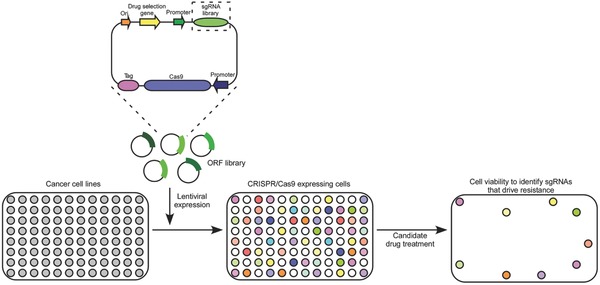
High‐throughput experimental approaches used in cancer drug‐ resistance studies. The top schemes represent the CRISPR/Cas9 expression vector, including sgRNA libraries (green). At the bottom, gain‐of‐function screen using ORF libraries to identify candidate drivers of resistance. Breast cancer cell targeting is conducted in multiwell plates using viral transduction. The readout is based on cell population measurement of individual wells after drug treatment.

Loss‐of function mutations mediated by the Cas9 endonuclease are achieved by generating a DSB in a constitutively spliced coding exon. Following that the DSB is repaired by NHEJ, it can introduce an indel mutation, which frequently leads to a coding frameshift for a premature stop codon and initiation of nonsense‐mediated decay of the transcript.^91^, ^92^, ^93^ Ruiz et al. identified CDC25A as an effector essential for resistance to Ataxia‐Telangiectasia and Rad3‐related (ATR) inhibitors in cancer therapy by using a CRISPR/Cas9 screening.^94^ A loss‐of‐function genetic screening approach, reported by Wang et al., was based on a pooled genome‐scale lentiviral sgRNA library to identify DNA mismatch repair pathway and identified genes relevant to resistance to DNA topoisomerase II poison etoposide.^92^ The genes have been identified whose loss confers resistance to the BRAF‐V600E inhibitor vemurafenib by using a positive selection screen with a pooled lentiviral library.^91^ Usually, the 5′ exons are preferred targets, as indels in these exons have a relative higher probability to introduce an early stop codon or a frameshift in the transcript of the protein. However, this approach may produce in‐frame variants that keep the functionality, which can obscure strong genetic dependencies. To overcome the limitation, Shi et al. targeted CRISPR/Cas9 mutagenesis to various exons encoding functional protein domains.^95^ This strategy could generate a higher proportion of null mutations, as well as significantly increases the potency of negative selection. Drug resistance screening based on Cas9 displays high reagent consistency, strong phenotypic effects, and high validation rates.^65^ Loss‐of‐function screening can identify genes that confer resistance to a specific drug when knocked out or knocked down (**Figure** **3**). Recently, Joung et al. described a protocol for genome‐scale knockout and screening for transcriptional activation by using the CRISPR/Cas9 system, which can be applied to the drug‐resistant gene screening in a relative short period.^96^

Gain‐of‐function screening is to identify genes that confer resistance to drug when overexpressed. Traditionally, a genome scale lentiviral expression library of human ORFs is used to reveal genes that drive drug resistance.^97^ It is apparent that such gain‐of‐function strategy can provide insights into resistance mechanisms to drugs that have not been approved for clinical studies.^88^ However, the gain‐of‐function screening has been limited to cDNA overexpression libraries that were incomplete because of the difficulty of cloning or expressing large site of cDNA constructs.^97^ The dCas9 system has an apparent advantage; that is, it mediates transcriptional activation that originates from the endogenous gene locus instead of exogenous cDNA construct. These libraries could be able to capture the full complexity of transcript isoforms instead of expressing genes independently of the endogenous regulatory context. To facilitate Cas‐9‐based gain‐of‐function screening, a synthetic activator could be constructed by fusing dCas9 with transcriptional activation domains, e.g., VP64 or p65.^98^ It has been shown that the delivery of multiple sgRNAs targeting at the same promoter region can improve target gene activation.^99^ A repeating array of peptide epitopes fused to dCas9 has been developed with activation effector domains to amplify the signal of dCas9 fusion effector domains. The Cas9 activation complex consists of a dCas9 fusion protein and modified sgRNA has been implemented for a genome‐wide gain‐of‐function screening to identify vemurafenib resistance in melanoma cells.^91^ Konermann et al. synthesized a library of 70 290 guides targeting full human RefSeq coding isoforms to screen genes that confer resistance to a BRAF inhibitor after activation.^100^

Immortalized cancer cell lines have been served as essential experimental models to study the genetic and biological properties of cancer in vitro. At present, there are more than 1000 cancer cell lines established and used globally, and substantial knowledge of drug resistance has been learned from studies on cancer cell lines. Although some breast cancer cell lines have been widely used and profiled, as well as drug‐resistant cell lines derived, it is increasingly essential that more genetic information is recapitulated. Meanwhile, patient‐derived drug‐resistant cells can also expand the repertoire of available drug‐resistance models. Patient‐derived tumor xenograft models are becoming more popular among researchers who need to get rid of the problem for prior selection in tissue culture.^101^ Cas9‐based drug‐resistant target screening can identify the players mediating drug resistance to substantially understand the complex biological processes and the large number of genes causing drug resistance, with the aid of genomic technologies.

Less than 2% of the mammalian genome encodes proteins, and most of the genome is transcribed into noncoding RNA (ncRNA).^102^ These transcripts play important roles in cellular metabolism and development, although the majority of them are yet to be studied thoroughly.^103^ Different classes of ncRNA are involved in human carcinogenesis.

A number of putative ncRNAs associated with poor overall survival may serve as prognostic markers for breast cancer, and dysregulation of copy number and expression is associated with cancer initiation and progression.^104^ Long noncoding RNAs (lncRNAs) play a critical role in cellular processes, ranging from transcriptional to post‐transcriptional regulation in breast cancer.^105^ Studies have shown that lncRNAs can control transcriptional alteration, with different lncRNA profiles in normal and cancer cells, which may be more than a secondary effect of cell transformation.^106^ For instance, the HOX transcript antisense RNA (HOTAIR) is transcribed from the HOXC locus on chromosome 12, the expression of which is increased in primary breast tumors and metastases, and HOTAIR expression level in primary tumors can be used as a powerful predictor of eventual metastasis and death.^107^ Several microRNAs, including miR‐200c and miR‐34a, have been shown to be closely linked to drug resistance in cancer and could be potential biomarkers for breast cancer diagnosis or therapy.^108^ Some RNAs are involved in the process of epithelial–mesenchymal transition, which is closely linked to drug resistance.^109^ To study ncRNAs, the most wildly used approach for gene functional studies is knockdown by RNAi, which is mainly functional in the cytoplasm where RNA‐induced silencing complex complexes are located. Nevertheless, many lncRNAs are localized in the nucleus, which makes it difficult to achieve robust knockdown. Thus, genetic editing such as CRISPR/Cas9 provides a better alternative at the genomic level because it targets the genomic DNA.^110^ Shechner et al. recently developed a platform called CRISPR‐Display to interrogate or repurpose ncRNA function.^111^ The CRISPR‐Display, which uses dCas9, could be applied to the investigation of the intrinsic functions of ncRNA by probing the contribution of the ncRNA to associated phenomena, including drug resistance in breast cancers. This genome editing based on the CRISPR/Cas9 system will be an essential tool for studying the functional and mechanistic role of ncRNA in breast cancer.

## 6 Limitations of CRISPR/Cas9 System Application

Along with our improved understanding of the mechanisms mediating drug resistance, it is important to select genes that offer the hope of delaying the development of resistance in the treatment of breast cancer. Thus, resistance target screening and identification, as well as reverting resistance gene mutations, with the CRISPR/Cas9 system may provide opportunities to mitigate drug resistance. However, within the CRISPR/Cas9 system, there are still some current limitations.

To improve target activity, thousands sgRNAs have been examined to establish numerous online tools to facilitate the selection of guide RNAs for specific sequences.^92^, ^112^ The crystal structure of CRISPR/Cas9 complex bound to target DNA has been solved, and variants of Cas9 protein have been engineered to improve the flexibility and precision of genome editing.^113^ Although the molecular mechanisms underlying high guide RNA efficiency are not completely understood, there are some prediction scores for guide activity that are available online to estimate its efficiency in targeting genes in different expression systems and species. To overcome the low editing efficiency of CRISPR/Cas9 for some specific loci, Cech and co‐workers has developed a “pop‐in/pop‐out” approach to enrich for the edited clones that have undergone HR and have been tagged, which could also be used for screening of effective gene modification, especially for loci hard to access.^114^

One of the concerns in employing CRISPR/Cas9 system for clinical therapy is the presence of antigen‐specific T‐cells directed against Cas9 protein. The immune reaction can eliminate gene‐edited cells that could lead to mortality, and recently Chew reviewed the potential immunological risk for CRISPR/Cas9 therapeutics toward clinic study.^115^ Another latest study shows the pre‐existing and adaptive immune response in humans cells against Cas9 proteins derived from the bacteria.^116^ It raises the potential problems to efficiently use, more importantly the safety, of the CRISPR/Cas9 system to treat disease. Shortening the expression of Cas9 or suppressing the immunity could temporarily prevent severe response during therapy if necessary. To eradicate the problem, however, more studies are required to identify and rule out the existence of SpCas9‐specific T‐cells during the therapy, or engineering recombinant Cas9 that can escape immune response.

Another important problem especially for clinical trial is the efficient and safe delivery of CRISPR/Cas9 into cell types or tissues that are hard to transfect and/or infect.^117^ Some nonviral delivery methods of CRISPR/Cas9 system have been used in studies in vitro or in vivo, including electroporation, injection, nanoparticles, or combinational methods. Nonviral methods have unique advantages over viral vectors delivery system, due to their transient expression patterns and the potential for repeated administration and advanced efficacy. However, only a few nonviral vectors and several physical methods have been used in the clinical research stages because of their own set of delivery challenges including large size and strong negative charges of the plasmid.^118^ A bacteriophage‐derived vector the vehicle that could be used to move CRISPR/Cas9 closer to clinical applications in a simple and efficient manner, but more preclinical studies must be implemented to test its potential genotoxic effects and evaluate the pharmacokinetic properties of phage‐derived nanoparticles as well as other undesired consequences. Recently CRISPR/Cas9 plasmid has been tried to be encapsulated into lipopolymer with cell specific aptamer for cancer targeted delivery.^119^ Wang et al. employed lipids and gold nanoclusters as platform for CRISPR/Cas9 system delivery to tumor cells. It not only shows higher efficiency than traditional transfection method, but effectively shows oncogene editing and tumor suppression in vivo.^120^ Meanwhile, another group designed liposome‐templated hydrogel nanoparticles for targeted delivery of CRISPR/Cas9 for cancer in vivo, and which can penetrate blood–brain barrier encouragingly.^121^ Besides the conventional methods, exosomes are also used as a platform to deliver CRISPR/Cas9 system in cancer cells efficiently.^122^ All these studies indicate promising targeted delivery system. However, even with a systemic delivery vector that can efficiently deliver CRISPR/Cas9 to cancer cells without obvious side effects, there is no guarantee that a full therapeutic effect will be achieved for specific type of cancer in clinical studies.^123^ A thoughtful understanding of the cancer cell drug process and connections between different cellular pathways in cancer cells is mandatory for developing efficient therapeutics, especially considering that each type of breast cancer has its own genomic and phenotypic profiles.^124^

There are current technical limitations to the use of CRISPR/Cas9 as a therapeutic strategy for targeting cancer genes in human patients. For instance, target site recognition by Cas9 requires the recognition of a short neighboring PAM. According to a recent study by Kleinstiver et al., engineered Cas9 derivatives with altered PAM specificities overcome this limitation based on structural information and combinatorial design.^125^ The SpCas9 PAM variants showed decent specificity and better discrimination against off‐target sites. This shows the feasibility of engineering Cas9 nucleases with new properties to improve the performance. Furthermore, Zetsche et al. reported the characterization of Cpf1, a RNA‐guided DNA nuclease that provides immunity in bacteria and could be adapted further for genome editing in mammalian cells.^126^ Following on this study, Fonfara et al. showed that Cpf1 from Francisella novicida cleaves upstream of a hairpin structure of pre‐crRNA in the CRISPR repeats and thereby generates intermediate crRNAs that are processed further, leading to mature crRNAs.^127^

Genetic screening implying the CRISPR/Cas9 technology can be performed with a library of sgRNA that targets the Cas9 endonuclease to specific loci. However, sgRNA libraries may not always cause a phenotype, when only typically target 5′ coding exons particularly with functional in‐frame variants are produced. To use CRISPR/Cas9‐induced mutagenesis identifying essential genes in a murine acute myeloid leukemia cell line, Shi et al. reported that the degree of negative selection varied greatly among sgRNAs targeting the same gene. They proposed a negative‐selection screening approach by using sgRNA libraries to target exons encoding potentially druggable protein domains which generated a higher proportion of null mutations improving the efficacy of negative selection.^95^ It may thus help to identify protein domains sustaining cancer cells and prioritize specific drug discovery.

Application of the CRISPR/Cas9 technique involves risks, such as off‐target mutations, that can be deleterious.^128^ The CRISPR/Cas9 system has to be carefully designed to avoid or decrease potential off‐target of cleavage sites, including with the mismatches to the 12 bases nearest to the guide strand PAM, and this is especially important in clinical oncology studies. In vitro, it has been shown that DNA annealing and cleavage of the guide to the target DNA could allow up to five mismatches.^56^ The low specificity raises an important concern for genome editing with CRISPR/Cas9 in living cells. This issue deserves thorough investigation when considering therapeutic applications. The off‐target cleavage events have been extensively examined, and a series of simple rules have emerged to minimize off‐target effects in research.^129^ Several methods have been employed to test off‐targets. Potential off‐target sites could be first identified with bioinformatics by searching the genome for sequences containing mismatches to the target that are followed by a PAM motif.^130^ Meanwhile, several online tools are available for guide RNAs selection to minimize off‐targets. Compared with the initial methods by the T7 assay, some more sensitive methods, including PCR amplicon sequencing, were demonstrated for evaluating off‐target mutations with an unbiased manner. All of these involved high‐throughput sequencing, such as GUIDE‐seq (genome‐wide, unbiased identification of DSBs enabled by sequencing), HTGTS (high‐throughput, genome‐wide, translocation sequencing), IDLV (integrase‐defective lentiviral vector), BLESS (direct in situ breaks labeling, enrichment on streptavidin and next‐generation sequencing), and Digenome‐seq (in vitro Cas9‐digested whole‐genome sequencing). A fair comparison for unbiased methods detecting off‐targets needs identical DNA samples from the same expression levels of guide RNA and Cas9, which could be very complicated because sensitivity is largely determined by the depth of high‐throughput sequencing. A conclusion from the studies that performed an unbiased detection for off‐targets effects is that the off‐targets detected are homologous to the guide in every case. Novel assays for genome‐wide off‐targets identification have provided crucial insights into the issue of cleavage specificity in vivo.

## 7 Perspectives and Concluding Remarks

CRISPR is a revolutionary gene editing strategy that has been rocking the world of biology ever since researchers realized they could apply it to modify the genome of any species with such ease and a precision never achieved before.^128^ Many recent studies have put this technology into application; for example, the gene‐edited monkey model has been established, as well as a mouse brain engineered with CRISPR/Cas9 system.^131^ It is a powerful tool and could be used to permanently alter the genome in a manner that could be passed on to future generations. The usage of CRISPR/Cas9 system revives many other social as well as ethical issues, not only for humans but also with other organisms and the environment, such as safety issues to avoid ecological impairment or the technique usage for genetic enhancement. More attention must be placed on risks; especially they may damage living beings and the environment. Meanwhile ethical concerns are raised regarding the possibility of genome editing of the human germline; that is, the genomic information that can be transmitted to following generations, from gametes, a fertilized egg, or first embryo divisions.^132^ Until now, all therapeutic interventions in humans employing genome editing have been performed in somatic cells. Liang et al. have created concern for the possibility of making changes within the human germline.^14^

So far, CRISPR/Cas9 system has not been applied to revert anticancer drug resistance in clinical studies. However, the emerging clinical trial has indicated that a gene‐editing technique could be safe and effective in humans. US and Chinese teams intended to use CRISPR/Cas9 system in similar ways, but on different types of cancers. The Chinese team is planning to target non‐small‐cell lung carcinoma; the US team will focus on melanoma, sarcoma, as well as myeloid cancers. Some scientists in China are on the verge of being the first in the world to inject patients with cells modified by the CRISPR/Cas9 gene‐editing technique.^133^ At the University of Pennsylvania, scientists are spearheading the small trial, hoping to use the technique to edit genes of the immune cells from patients, reprogramming them for recognizing and attacking cancer at the first signs of growth.^134^ In conclusion, traditional approaches applied to drug development may be inappropriate for new targeting agents. Resistance to many traditional and new drugs is a major clinical challenge for cancer treatment. The use of specific targeting technologies will lead us to understand the mechanisms of signaling pathways as the roads of the “genomic landscape” of breast cancer.^2^ Further insight into the molecular mediators of resistance will have a great impact on the ability to target genes or pathways that could overcome drug resistance for improving clinical outcomes. Therefore, although it still exists of technical limitations to the usage of CRISPR/Cas9 system for targeting cancer genes in human patients, the prospects of gene therapy are nonetheless very exciting. CRISPR‐based genome editing will serve as a critical tool for both bench and bedside. Carefully designed sgRNA, well management of the potential off‐target effects, and efficient delivery will be the essential for the success of the CRISPR/Cas9‐mediated therapy. The development of this technology from basic research to clinical application provides exciting opportunities for understanding and treating drug resistance. In the era of personalized medicine, CRISPR/Cas9‐based approaches will become an improved strategy to tackle the complexity of various cancers and cancer drug resistance, which is the ultimate goal of precision medicine.

## Conflict of Interest

The authors declare no conflict of interest.
